# Overexpression of GmHsp90s, a Heat Shock Protein 90 (Hsp90) Gene Family Cloning from Soybean, Decrease Damage of Abiotic Stresses in *Arabidopsis thaliana*

**DOI:** 10.1371/journal.pone.0069810

**Published:** 2013-07-25

**Authors:** Jinyan Xu, Chenchen Xue, Dong Xue, Jinming Zhao, Junyi Gai, Na Guo, Han Xing

**Affiliations:** Key Laboratory of Biology and Genetics and Breeding for Soybean/National Key Laboratory of Crop Genetics and Germplasm Enhancement, Ministry of Agriculture/National Center for Soybean Improvement, Nanjing Agricultural University, Nanjing, P.R. China; Virginia Commonwealth University, United States of America

## Abstract

Hsp90 is one of the most conserved and abundant molecular chaperones and is an essential component of the protective stress response; however, its roles in abiotic stress responses in soybean (*Glycine max*) remain obscure. Here, 12 *GmHsp90* genes from soybean were identified and found to be expressed and to function differentially under abiotic stresses. The 12 GmHsp90 genes were isolated and named *GmHsp90A1–GmHsp90A6*, *GmHsp90B1*, *GmHsp90B2*, *GmHsp90C1.1*, *GmHsp90C1*.*2*, *GmHsp90C2*.*1* and *GmHsp90C2*.*2* based on their characteristics and high homology to other Hsp90s according to a new nomenclature system. Quantitative real-time PCR expression data revealed that all the genes exhibited higher transcript levels in leaves and could be strongly induced under heat, osmotic and salt stress but not cold stress. Overexpression of five typical genes (*GmHsp90A2*, *GmHsp90A4*, *GmHsp90B1*, *GmHsp90C1.1* and *GmHsp90C2*.*1*) in *Arabidopsis thaliana* provided useful evidences that GmHsp90 genes can decrease damage of abiotic stresses. In addition, an abnormal accumulation of proline was detected in some transgenic Arabidopsis plants suggested overexpressing GmHsp90s may affect the synthesis and response system of proline. Our work represents a systematic determination of soybean genes encoding Hsp90s, and provides useful evidence that GmHsp90 genes function differently in response to abiotic stresses and may affect the synthesis and response system of proline.

## Introduction

Since plants are immobile, they must cope with various acute environmental changes such as extreme temperature, salt and osmotic stresses. Proteins can be misfolded and aggregate under stress and lead to many problems in a cell, and so all organisms have dedicated protein assemblies that maintain proteostasis and mitigate the life-threatening effects of stresses on the proteome [1]. At the core of this repertoire are molecular chaperones and protein remodeling factors of which folding agents constitute a large, diverse and structurally unrelated group. Many are up-regulated in response to heat and are therefore termed heat shock proteins (HSPs) [1]. Hsp90 is one of the most conserved and abundant proteins, presents from bacteria to mammals, and is an essential component of the protective stress response [1]. In addition, it is also involved in signal transduction [2], [3], [4], cell-cycle control [5], protein degradation [6] and protein trafficking [7], [8] and acts as a capacitor of phenotypic variation in *Arabidopsis thaliana* [9]. Because of its importance it has been extensively studied in many species but little in plants.

In plants Hsp90 have been identified from Pharbitis nil [10], Brassica napus [11], [12], Secale cereale [13], Zea mays [14], Hordeum vulgare [15], Arabidopsis thaliana [16], [17], [18], Oryza sativa [19], [20], [21], Glycine max [22] and Vitis vinifera [23], and most are strongly induced by various abiotic stresses.

In eukaryotes all Hsp90 genes are located on the nuclear genome, but their proteins function in the cytoplasm, endoplasmic reticulum (ER), chloroplasts and mitochondria. Chen *et al.* [24] divided the gene family into five subfamilies, HSP90A, HSP90B, HSP90C, TRAP and HTPG and established a new nomenclature system based on the phylogeny of their proteins and the cell compartments in which the proteins function. In Arabidopsis the Hsp90 family includes seven members. The *AtHsp90-1*–*AtHsp90*-4 proteins constitute the cytoplasmic subfamily, whereas the *AtHsp90*-5, *AtHsp90*-6 and *AtHsp90*-7 proteins are within the chloroplast, mitochondria and ER compartments, respectively [16].

Transcripts of *AtHsp90.1 *can be detected only in roots of control Arabidopsis plants but are abundant in all organs after heat shock or treatment with heavy metals; whereas *AtHsp90*.2, *AtHsp90*.3 and *AtHsp90*.4 are constitutively expressed [16], [25]. Transcripts of *AtHsp90.5* are mildly induced by heat shock, and *AtHsp90.6* is barely induced by heat shock [16], [26]. Expression of three organellar and two cytoplasmic AtHsp90s (*AtHsp90.1* and *AtHsp90*.2) in a temperature-sensitive Hsp90 mutant and a conditional Hsp90-null mutant of yeast (*Saccharomyces cerevisiae*) showed that cytoplasmic *AtHsp90*.1 and *AtHsp90*.2 functioned similarly to that of yeast in chaperoning roles [27]. They could support the growth of yeast mutants at both permissive and non-permissive temperatures; however, neither the full-length nor mature forms of chloroplast-located *AtHsp90*-5, mitochondria located *AtHsp90*.*6* nor ER-located *AtHsp90*.*7* could complement the yeast Hsp90 proteins. This indicates that organellar and cytoplasmic AtHsp90 possibly work through different molecular mechanisms [27].

Soybean [*Glycine max* (L.) Merr.] is an important crop plant, and its yield is severely affected by various environmental conditions, such as heat stress or osmotic stress. In soybean, two Hsp90s (*GmHSP90-1* and *GmHSP90-2*) have been cloned by Fu *et al*. [22]; However, their roles in abiotic stresses remain obscure. In this study, we report the identification of 12 Hsp90 genes from soybean. For ease of study, we use a new nomenclature system and name them *GmHsp90A1–GmHsp90A6*, *GmHsp90B1*, *GmHsp90B2*, *GmHsp90C1*.*1*, *GmHsp90C1*.*2*, *GmHsp90C2*.*1* and *GmHsp90C2*.*2* based on their characteristics and high homology to other Hsp90s. Their expression patterns in different tissues and abiotic stresses were characterized. Five typical genes (*GmHsp90A2*, *GmHsp90A4*, *GmHsp90B1*, *GmHsp90C1*.1 and *GmHsp90C2*.1) whose homology and expression patterns differ were further characterized for their function in abiotic stress responses using an overexpression approach in model plant Arabidopsis. These studies are significant in elucidation of the role of GmHsp90 in response to abiotic stresses.

## Materials and Methods

### Plant Materials, Growth Conditions and Stress Treatment

Soybean (*G. max* L.) cv. Qihuang22 was used to isolate GmHsp90 genes and to examine their expression patterns. For tissue-specific expression analysis, seeds were field-grown under standard agronomic practices at Nanjing Agricultural University and roots, stems, leaves, flowers and developing seeds were collected. For osmotic and salt treatments, three-week-old seedlings grown at 28/25°C with a 12/12 h (light/dark) photoperiod in an artificial climate box were saturated in water (control), PEG (20%) or NaCl (200 mM), respectively. For heat and cold treatments, soybean seedlings were saturated in water and then moved to 42°C or 4°C with a 12/12 h (light/dark) photoperiod in an artificial climate box. Leaves from all the treatments above were harvested at 0, 0.5, 1, 3, 6, 12 and 24 h then stored at –70°C for RNA extraction.

Arabidopsis plants used for transformation was grown in an artificial climate box at 22/20°C with a 16/8 h (light/dark) photoperiod. Seeds from control or homozygous transgenic plants were sown on quartz sand and filter paper saturated with water (control), mannitol (350 mM) or NaCl (150 mM), respectively, for osmotic and salt treatments. For heat treatments, seeds were also sown on quartz sand and filter paper saturated with water and then altered to 30°C. Germination rate from all treatments above were calculated.

Seedlings of control and homozygous transgenic plants were grown in a greenhouse at 22/20°C with 16/8 h (light/dark) photoperiod. For heat stress, three-week-old seedlings were altered to 30°C in a greenhouse until pod setting. Rosette leaf samples were taken after 3 d of stress. Shoot fresh weights were measured when pod setting was calculated. For osmotic and salt treatments, three-week-old seedlings were saturated with water (control), PEG (8%) or NaCl (150 mM), respectively. Rosette leaf samples were taken at 3 d after treatment. Control plants corresponding to stress treatments were treated with the same conditions. The leaf discs of three-week-old transgenic and control plants were saturated with water and exposed to a temperature of 65°C for 15 min relative electrical conductivity was measured.

### Isolation and Sequence Analysis of GmHsp90s

To identify putative Hsp90 genes in soybean, *GmHsp90-1* (FJ222390) and *GmHsp90*-2 (FJ222389) [22] were BLAST searched at http://www.phytozome.net/soybean [28]. Total RNA was isolated from seedlings of soybean cv. Qihuang22 using an RNAprep pure Plant Kit (Tiangen) and cDNA was prepared using the Prime Script RT-RCP Kit (TAKARA). The full -length cDNA was obtained using gene-specific primers. The PCR products were inserted into pMD19-T simple vector (TAKARA) and then sequenced (Invitrogen, Shanghai). Subcellular localization of the deduced polypeptides was predicted by WoLF SPORT (http://wolfpsort.org) [29], [30] and TargetP 1.1 Server (http://www.cbs.dtu.dk/services/TargetP) [31]. Molecular weight (MW) and theoretical pI (isoelectric point) of polypeptides was determined using Compute pI/MW tool (http://web.expasy.org) [32]. Multiple-aligned sequences were determined by ClustalX [33], and GeneDoc [34] was used to manually edit the results. Phylogenetic analysis was conducted using the neighbor-joining algorithm included in MEGA4.0. software [35]. The Hsp90 gene family signature was detected by scanning sequences against PROSITE patterns and profiles at http://web.expasy.org [32].

### Quantitative Real-time PCR Analysis

Total RNA was extracted using an RNAprep pure Plant Kit (Tiangen) and isolated RNA was subjected to reverse transcription using the ReverTra Ace® qPCR RT Kit (TOYOBO, Japan). Quantitative real-time PCR was performed on each cDNA template using the iQ SYBR Green Supermix (Bio-Rad, Singapore) on a Bio-Rad iQ5 real-time PCR system. The specificity of the reactions was verified by melting curve analysis. The relative mRNA level for each gene was calculated as ΔΔC_T_ values [36], [37]. Soybean beta *tubulin* gene was used as internal control for normalization. The result of tissue-specific expression was quantified and the values obtained were analyzed using the Cluster 3.0 program [38]. The results were displayed with TreeView [39]. The result of abiotic stress expression was processed as relative expression fold and was generated by SigmaPlot 9.0 [40].

### Construction of GmHsp90 Plant Expression Vectors and Plant Transformation

We used a gateway technology (Invitrogen, USA) to construct the vector and pEarleyGate103 [41] was used as the destination vector. The pEarleyGate103 -35S-GmHsp90 plasmids were constructed by amplifying the coding region of GmHsp90 through PCR with the primers in Table S4. Arabidopsis plants were transformed by the floral dip method [42] using *Agrobacterium tumefaciens* strain EHA105. T_1_ seeds were collected and selected on MS medium [43] containing 20 mg/L Basta and then tested by PCR. T_3_ or T_4_ homozygous lines were used for further study. We also transformed the vector pEarleyGate103 into Arabidopsis and the homozygous lines named as control plants.

### Physiological Indices Test

Free proline content was determined using the ninhydrin method of Bates *et al*. [44]. Arabidopsis plants were homogenized in 4 ml of sulfosalicylic acid (3%) and the homogeneous mixture was placed in boiling water for 10 min. After cooling, the mixture was filtered through filter paper. The extract (1 ml) was added to a new tube and mixed with 1 ml of sulfosalicylic acid (3%), 1 ml of acetic acid and 2 ml of acid ninhydrin. The reaction mixture was boiled for 1 h and cooled in an ice bath and then 2 ml of toluene was added to the extract and thoroughly mixed. Finally 150 µl of the toluene extract was removed for absorbance measurement at 520 nm in a TECAN M200 Microplate reader. A standard curve was drawn using proline (Sigma, USA).

Lipid peroxidation was determined by measuring malondialdehyde (MDA) formation using the thiobarbituric acid method [45], [46] and partly according to GB/T.181-2003. Arabidopsis plants were homogenized with 10% trichloroacetic acid (TCA). The homogenate was centrifuged at 12,000×g for 20 min. For every 0.8 ml of the extract, 2 ml of 0.5% thiobarbituric acid (TBA) containing 10% TCA was added. The mixture was boiled in a water bath for 15 min and then cooled. Of the final extract, 150 µl was removed for absorbance measurement at 600, 450 and 532 nm in a TECAN M200 Microplate reader. A standard curve was drawn with 1,1,3,3-tetraethoxypropane (Sigma, USA).

Total chlorophyll was extracted with 80% acetone. After filtering, absorbance of 150 µl of clear chlorophyll solution was measured at 663, 645 and 750 nm in a TECAN M200 Microplate reader. Total chlorophyll was estimated according to Porra *et al.* [47].

Membrane permeability of leaves under stress was measured by electrolyte leakage, which was measured as relative electrical conductivity [48], [49]. Electrical conductivity of Arabidopsis leaves was assayed using an electronic conductivity meter (FE30, Mettler Toledo). Electrical conductivity of ultra-pure water was recorded as L_0_. Leaves of every treatment group were immersed in 20 ml of ultra-pure water and the measured electrical conductivity recorded as L_1_. After continuous shaking in darkness for 24 h, electrical conductivity was measured again and recorded as L_2_. Relative electrical conductivity was estimated as (L_1_– L_0_)/(L_2_– L_0_).

### Statistical Analysis

Statistical analysis of the experimental data was conducted by ANOVA with SPSS [50].

## Results

### Isolation and Sequence Analysis of Hsp90 Genes from Soybean

As the whole soybean genome sequence was completed in 2008 [28], we used two GmHsp90 genes, *GmHsp90*-1 (FJ222390) and *GmHsp90*-2 (FJ222389) [22], to BLAST against the soybean genome. After excluding predicted genes with no consistent homology to Hsp90s or with an incomplete open-reading frame (ORF), 12 putative Hsp90s were retained for further analysis. Base on the sequence obtained, specific primers of each sequence for the full-length candidate genes were designed (Table S1). We also used the primers of Fu *et al.* (2009) to isolate the *GmHsp90*-1 (FJ222390) and *GmHsp90*-2 (FJ222389). The sequences we obtained were slightly different to those published in NCBI but the same as *GmHsp90*-8 and *GmHsp90*-9. It is possible that they were from different soybean varieties. We used the sequences isolated from cv. Qihuang22 for further analysis.

The soybean HSP90 family is distributed on different chromosomes and the sizes of their ORFs were in the range of 2097–2447 bp, while MW was 80.13–93.56 kDa (Table 1). We compared the predicted amino acid sequence similarity among all 12 members of the Hsp90 family, and this indicated that they could be divided into four groups according to their similarity (Table S2). Members of the same group had high homology but lower similarity to other groups. The homology between GmHsp90-7, GmHsp90-8, GmHsp90-9 and GmHsp90-13 was almost 98.4%, while GmHsp90-4 and GmHsp90-12 had about 97.4% homology to each other but only about 50% to other groups. GmHsp90-3 and GmHsp90-11 had almost 97% homology but only 65% homology to GmHsp90-6 and GmHsp90-14, and only had 44% similarity to other groups.

**Table 1:** The nomenclature of the 12 GmHsp90 genes and protein information.

Locus ID	Gene nomenclature	ORF size (bp)	Size (aa)	pI	MW (kDa)
Glyma09g24410	GmHsp90A1	2097	699	4.69	80.4
Glyma16g29750	GmHsp90A2	2097	699	4.7	80.34
Glyma08g44590	GmHsp90A3 (GmHsp90-1)	2097	699	4.69	80.13
Glyma14g01530	GmHsp90A4 (GmHsp90-2)	2100	700	4.77	80.16
Glyma02g47210	GmHsp90A5	2106	702	4.7	80.22
Glyma18g08220	GmHsp90A6	2100	700	4.67	80.2
Glyma14g40320	GmHsp90B1	2448	816	4.6	93.56
Glyma17g37820	GmHsp90B2	2442	814	4.59	93.3
Glyma01g09310	GmHsp90C1-1	2379	793	4.65	90.14
Glyma02g13790	GmHsp90C1-2	2382	794	4.63	90.05
Glyma02g47580	GmHsp90C2-1	2373	791	5.04	89.69
Glyma14g01100	GmHsp90C2-2	2391	797	4.88	90.57

### Characteristics and Phylogenetic Analysis of the Hsp90 Family Proteins from Soybean

**Figure 1:**
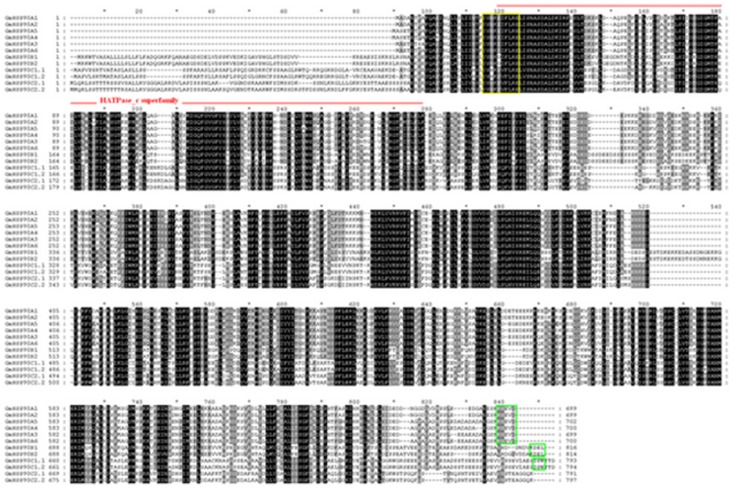
Multiple sequence alignment of predicted soybean HSP90 protein. Multiple-aligned sequences were determined by ClustalX, and GeneDoc was used to manually edit the results. Identical or similar acids are shown in black or gray, respectively. The HATpase_c signature sequence (red line) and signature motif ‘Y-x-[NQHD]-[KHR]-[DE]-[IVA]-F-[LM]-R-[ED]’ (yellow rectangle drawn) of Hsp90 were detected by scanning sequences against PROSITE patterns and profiles at http://web.expasy.org. The motif MEEVD, KDEL, DPW which is diagnostic of cytoplasmic, ER-retention, chloroplast and mitochondrial Hsp90, respectively, was bounded by a green rectangle drawn.

For further analysis, multiple sequence alignments were done; 12 GmHsp90s identified were highly conserved as for other species and possessed the Hsp90 family signature motif ‘Y-x-[NQHD]-[KHR]-[DE]-[IVA]-F-[LM]-R-[ED]’ and the ATP binding domain close to the N-terminal region (Fig. 1) [51], [52]. As expected, they were clearly distinguished as four groups, with the exception of the first six sequences being highly conserved, and each group had two highly conserved members (Fig. 1).

**Figure 2:**
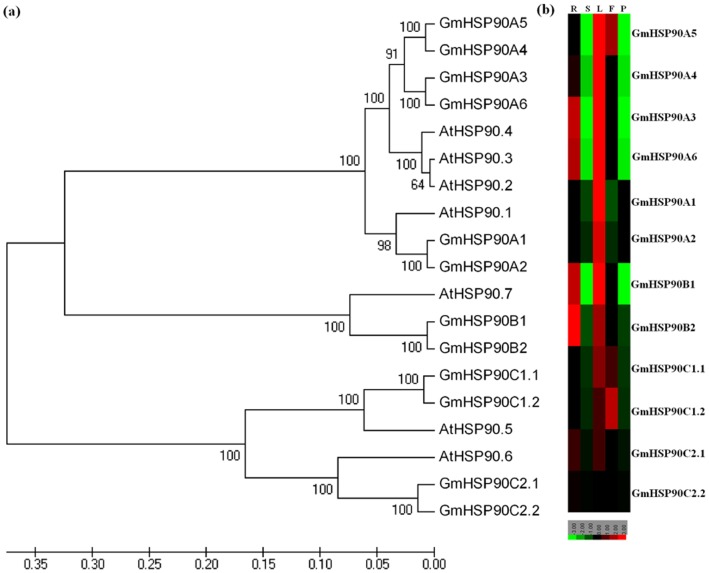
Phylogenetic relationships among *Glycine max* (GmHsp90) and *Arabidopsis thaliana* (AtHsp90) and expression of GmHsp90 genes in various soybean tissues. (a) Phylogenetic relationships among *G. max* (GmHsp90) and *A. thaliana* (AtHsp90). The trees were constructed using the neighbor-joining algorithm included in the MEGA4.0. software. (b) Expression of GmHsp90 genes in various soybean tissues. Quantitative real-time PCR was used to analyze the relative transcript levels of 12 GmHsp90 genes in various tissues. The results were each calculated from three technical repeats and then quantified; the values obtained were analyzed using the Cluster 3.0 program and displayed with TreeView. Samples of each repeat were prepared from at least five independent plants. There are significantly higher transcripts levels in the leaves, and the genes from the same branch have similar expression patterns. R, root; S, stem; L, leaf; F, flowers; P, pod 10 day after flower.

The phylogenetic relationships of soybean Hsp90 genes with those of Arabidopsis were investigated. Like the Hsp90 genes from Arabidopsis, all genes from soybean formed three distinct clades, and genes in the same clade had high homology (Fig. 2a). Based on the results of multiple sequence alignments (Fig. 1), phylogenetic relationships (Fig. 2a, S1), the new nomenclature system for Hsp90 [24]and subcellular localization prediction, as well as for ease of study of the GmHsp90 genes, we renamed the 12 GmHsp90s (Table 1). GmHsp90 proteins included a C-terminal pentapeptide MEEVD (Fig. 1), which is diagnostic of cytoplasmic Hsp90 proteins from both plants and animals [24], [53] were named GmHsp90A1–GmHsp90A6. As GmHsp90-1 and GmHsp90-2 were similar to GmHsp90-8 and GmHsp90-9, respectively, and we did not find more complete sequences in the database, we considered that they were the same genes and named them GmHsp90A3 and GmHsp90A4, respectively. GmHsp90-4 and GmHsp90-12 had high homology to ER Hsp90s and contained a putative C-terminal KDEL ER-retention motif (Fig. 1, S1a), indicating they may be ER Hsp90 proteins [24], [53]. So we named them GmHsp90B1 and GmHsp90B2, respectively. Chen *et al.* [24] named the high homology chloroplast and mitochondrial Hsp90 sequence containing a DPW motif at their C-terminal as HSP90C1, and the copies with high similarity to HSP90C1 but lacking the DPW motif as HSP90C2– thus we named the last four sequences in Fig. 1 GmHsp90C1.1, GmHsp90C1.2, GmHsp90C2.1 and GmHsp90C2.2 which had high homology to chloroplast AtHsp90-5 and mitochondrial AtHsp90-6 [16], [54], respectively (Fig. 1, S1b).

The phylogenetic tree indicated that, in the first clade formed by HSP90A, there were two well- defined branches which separated GmHsp90A1 and GmHsp90A2 from other GmHsp90A (Fig. 2a). GmHsp90A3–GmHsp90A6 and AtHsp90-2–AtHsp90-4 were so similar that we could not separate them further and showed that cytoplasmic HSP90 had high homology, whether from soybean or Arabidopsis. GmHsp90A1 and GmHsp90A2 had lower homology with other GmHSP90A– perhaps there is some functional difference between them. HSP90A and HSP90B had higher homology than HSP90C, as if they had originated from different ancestors [13].

Multiple sequence alignments and phylogenetic analysis showed that the numbers of Hsp90 genes in soybean were almost twice that in Arabidopsis and were in pairs. Soybean is a well-known palaeopolyploid, and we consider that Hsp90 genes were duplicated during a soybean duplication event. However, Arabidopsis has seven Hsp90 genes and we only obtained 12 Hsp90 genes in soybean. When searching the soybean genome database we found two other sequences with high homology to Hsp90 genes but they were much shorter and did not have complete ORFs. We consider there may be some mistakes in the duplication process of the two sequences leading to loss of their fragments, and so there remain only 12 normal Hsp90 genes.

### Expression of GmHsp90 Genes in Various Soybean Tissues

To analyze the expression of soybean HSP90 genes in different soybean tissues, quantitative real-time PCR was performed for 12 GmHsp90 genes (Fig. 2b). Gene-specific primers were also designed for each member (Table S3). Genes from the same branch (Fig. 2a) had more similar expression patterns, indicating that they may be functionally redundant. Although expression was diverse in different branches, there were significantly higher transcript levels in the leaves. It may be that the leaves are always exposed to high temperature during soybean cultivation, so GmHsp90 genes accumulate in leaves for more protection. Some branches had the same expression pattern in various soybean tissues but different transcript levels, suggesting a major role of higher transcript level genes in soybean growth, such as *GmHsp90B1* and *GmHsp90B2*. *GmHsp90C1* were more highly transcribed in flowers, while *GmHsp90A3*, *GmHsp90A6* and *GmHsp90B* genes were expressed more highly in roots than other genes, indicating that they may play some different roles in soybean growth.

### Expression of GmHsp90 Genes under Different Abiotic Stresses

**Figure 3:**
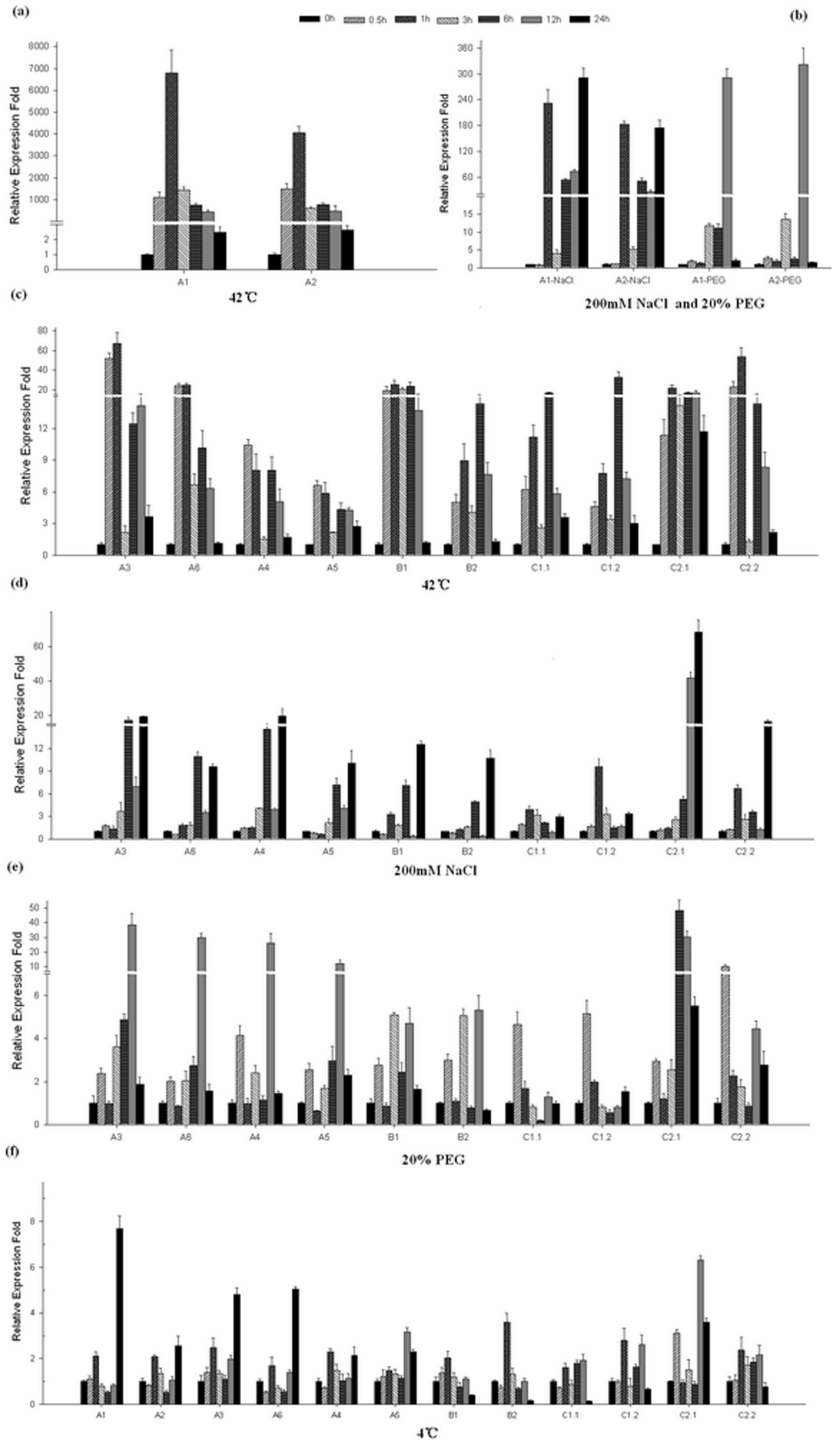
Quantitative real-time PCR analyses of the relative expression fold of 12 GmHsp90 genes under different stresses in soybean. Leaves of soybean from all the treatments harvested in 0, 0.5, 1, 3, 6, 12 and 24 h were used for the analysis. A soybean *beta tubulin* gene was used as internal control for normalization and the relative mRNA level for each gene was calculated as ΔΔC_T_ values. The result of expression was processed as relative expression fold and was generated by SigmaPlot 9.0. The transcript levels (means ± SD) displayed were each calculated using the qRT- PCR results of three technical repeats. Leaves of each repeat were prepared from at least five independent plants. (a) Relative expression folds of *GmHsp90A1* and *GmHsp90A2* under heat shock. (b) Relative expression folds of *GmHsp90A1* and *GmHsp90A2* under salt and osmotic stress. (c) Relative expression folds of the remainder of GmHsp90 genes under heat stress. (d) Relative expression folds of the remainder of GmHsp90 genes under salt stress. (e) Relative expression folds of the remainder of GmHsp90 genes under osmotic tress. (f) Relative expression folds of GmHsp90 genes under cold stress. A1, *GmHSP90A1*; A2, *GmHSP90A2*; A3, *GmHSP90A3*; A4, *GmHSP90A4*; A5, *GmHSP90A5*; A6, *GmHSP90A6*; B1, *GmHSP90B1*; B2, *GmHSP90B2*; C1.1, *GmHSP90C1.1*; C1.2, *GmHSP90C1.2*; C2.1, *GmHSP90C2.1*; C2.2, *GmHSP90C2.2.*

To further understand the function of GmHsp90 genes in soybean, expression of GmHsp90 under several stresses was analyzed at the transcript level (Fig. 3). A significant up- regulation was observed as early as 30 min at 42°C among all GmHsp90 genes and then decreased to the normal level at 24 h (Fig. 3a, c). *GmHsp90A1* and *GmHsp90A2* were strongly induced upon heat stress and the relative expression of the two genes increased up to about 6000 or 4000 times after 1 h at 42°C, thereafter transcripts decreased but remain at elevated levels compared to other genes until 24 h. All the GmHsp90A genes had the highest transcript levels at 1 h, indicating their rapid response to heat stress. GmHsp90B and GmHsp90C genes were also strongly induced by heat stress but the highest transcript levels were delayed compared with GmHsp90A. Similar to tissue- specific expression, *GmHsp90B1* and *GmHsp90C2*.1 had relatively high and continuing transcript levels compared to *GmHsp90B2* and *GmHsp90C2*.2 during heat stress – further suggesting their major role.

GmHsp90 expression also responded to salt stress, especially *GmHsp90A1* and *GmHsp90A2* (Fig. 3b, d). The response to salt stress was not as rapid as for heat stress. As well as *GmHsp90A1*, *GmHsp90A2* and *GmHsp90C1*, the expression of GmHsp90 genes was significantly up-regulated at 3 or even 6 h after stress. Generally, the entire peaks had appeared by 24 h, except for *GmHsp90C1*.

Like heat and salt stresses, GmHsp90 genes were also strongly induced by osmotic stress, although the transcript levels of GmHsp90B and *GmHsp90C1* were slightly lower (Fig. 3b, e). Unlike heat and salt stress, the response of *GmHsp90A1* and *GmHsp90A2* to osmotic stress was delayed to 3 h, while the expression of other genes was slightly raised at 1 h and all peaks had appeared by 12 h (except for GmHsp90C) and decreased abruptly to normal levels at 24 h. The expression pattern differed between *GmHsp90C2*.*1* and *GmHsp90C2*.*2*, suggesting there may be some functional complementation under osmotic stress. The GmHsp90 genes responded to osmotic stress more quickly than to salt stress but more slowly than to heat stress. However, we did not detect high up-regulation under cold stress but down- regulation at some time points (Fig. 3f).

GmHsp90 genes were induced by heat, salt and osmotic stresses but the response times and expression abundances were diverse. Moreover there were obvious differences between *GmHsp90A1* and *GmHsp90A2* and other GmHsp90A genes. These results suggest these genes may have different responses to the different stresses.

Overexpression of five GmHsp90 genes in Arabidopsis plants and germination rate of the seeds from overexpressing plants under different stresses.

To further understanding the function of GmHsp90 under abiotic stresses, five typical genes (*GmHsp90A2*, *GmHsp90A4*, *GmHsp90B1*, *GmHsp90C1*.*1* and *GmHsp90C2*.*1*) whose homology and expression patterns differ (Fig. 1–3) were further characterized using an overexpression approach. Transgenic Arabidopsis plants overexpressing each of the five genes were generated and the genes were under control of a cauliflower mosaic virus (CaMV) 35S promoter. For each gene, at least 15 transgenic lines were obtained, and two homozygous lines with the highest expression of the transgenic plants were analyzed further (Fig. S2).

**Figure 4:**
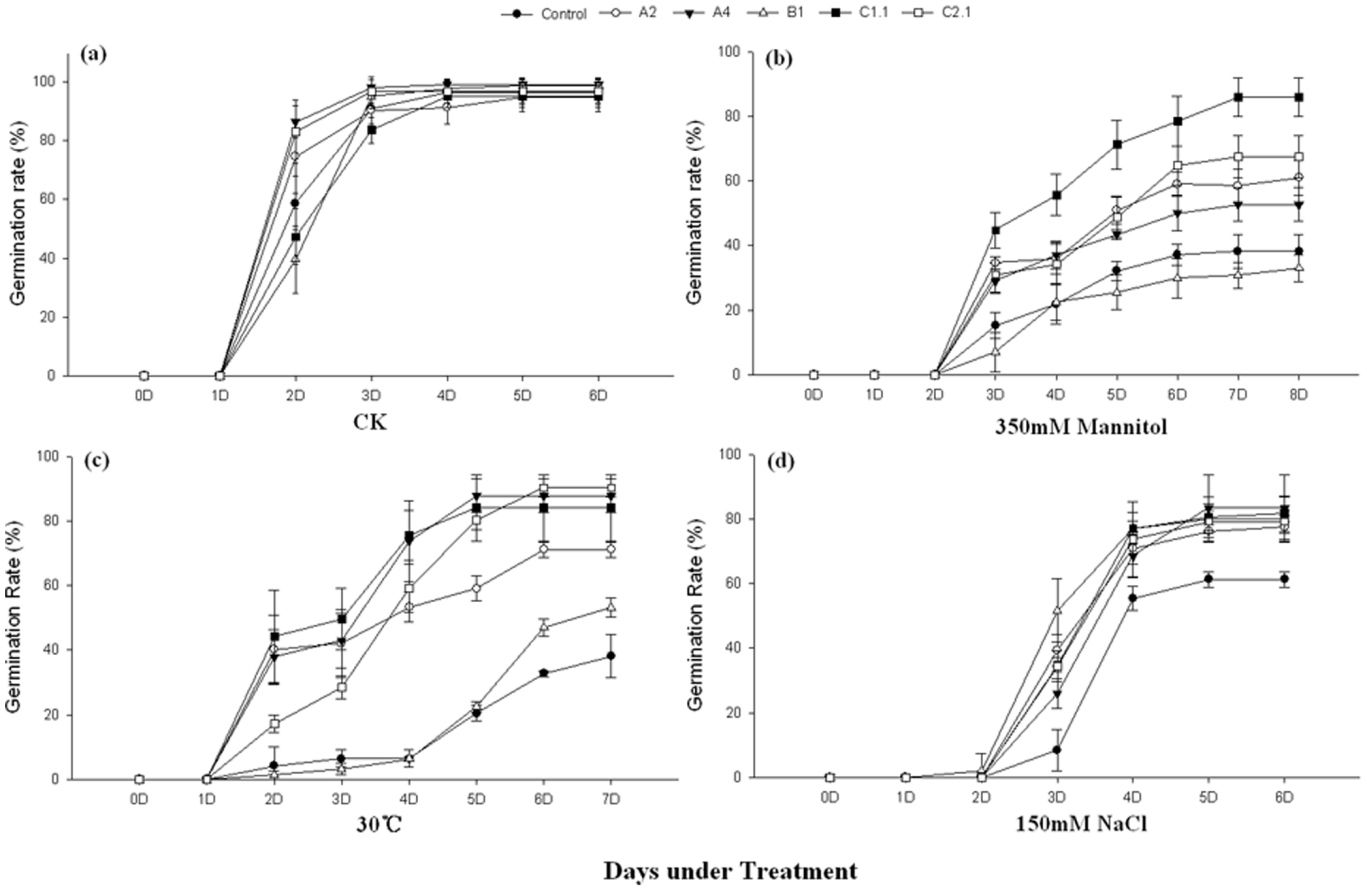
Seed germination rates of GmHsp90 overexpressing Arabidopsis and control plants under different stress. Seeds from control or homozygous transgenic plants were sown on quartz sand and filter paper saturated with water (control), mannitol (350 mM) or NaCl (150 mM), respectively, for osmotic and salt treatments. For heat treatments, seeds were also sown on quartz sand and filter paper saturated with water and then moved to 30°C. (a) Seed germination rates under normal conditions. (b) Seed germination rates under osmotic stress. (c) Seed germination rates under heat stress. (d) Seed germination rates under salt stress. Error bars indicate SD; n = 4. Control, vector control plants; A2, A4, B1, C1.1, C2.1 represent the *GmHsp90A2*, *GmHsp90A4*, *GmHsp90B1*, *GmHsp90C1*.1 and *GmHsp90C2*.1 transgenic lines, respectively.

We first analyzed the phenotype of the overexpressing Arabidopsis plants under abiotic stresses by calculating the germination rates (Fig. 4). In normal conditions the overexpressing and vector control seeds (control) showed no obvious differences in final germination (Fig. 4a). Most seeds started to germinate on the second day. When treated with heat, NaCl or mannitol, the germination of all seeds was impaired. The germination time was delayed in all seeds under salt and osmotic but not heat stress (Fig. 4). However, the germination rates of overexpressing seeds were higher than controls, except for *GmHsp90B1* (Fig. 4b–d). There were no significant differences between *GmHsp90B1* overexpressing seeds and controls under osmotic stress and the final germination rate of *GmHsp90B1* overexpressing seeds was slightly higher than controls but still signifycantly lower than other test seeds under heat stress. Overall GmHsp90 overexpressing Arabidopsis showed less germination rates decrease than control under heat, salt and osmotic stresses.

### Overexpressing GmHsp90 Affected Growth and Development of Arabidopsis under Abiotic Stresses

**Figure 5:**
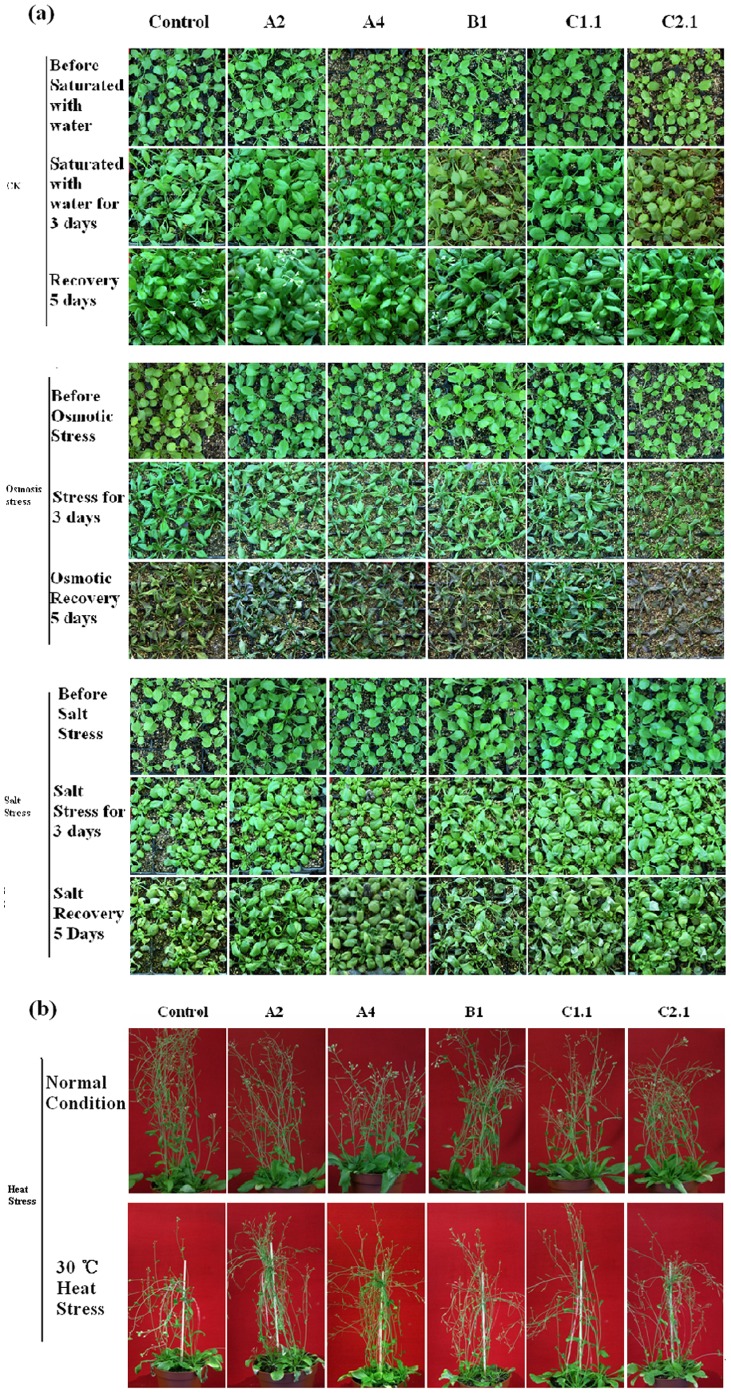
The growth of transgenic and control Arabidopsis plants under normal or abiotic stresses conditions. Three-week-old seedlings were saturated with water (control), PEG (8%) or NaCl (150 mM) respectively for 3 d and recovered for 5 d. For heat stress, three-week-old seedlings were moved to 30°C until pod setting. (a) Phenotype of Arabidopsis under normal conditions and osmotic or salt stress. (b) Phenotype of Arabidopsis under normal conditions and heat stress. Control, vector control plants; A2, A4, B1, C1.1, C2.1 represent the *GmHsp90A2*, *GmHsp90A4*, *GmHsp90B1*, *GmHsp90C1*.1 and *GmHsp90C2*.1 transgenic lines, respectively.

**Figure 6:**
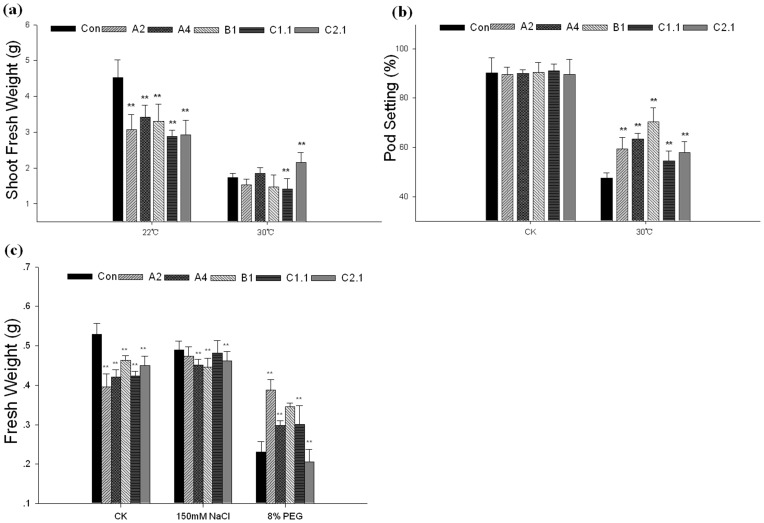
Fresh weight and pod setting percentage of transgenic Arabidopsis plant under normal or abiotic stresses. Three-week-old seedlings were saturated with water (control), PEG (8%) or NaCl (150 mM) respectively for 3 d and recovered for 5 d. Fresh weights were measured after 3 d of treatment. For heat stress, three-week-old seedlings were moved to 30°C until pod setting. Shoot fresh weights were measured when pod setting was calculated. (a) Shoot fresh weight of Arabidopsis under heat stress. (b) Pod setting percentage of Arabidopsis under heat stress. (c) Fresh weights of Arabidopsis plants under normal condition, salt and osmotic stress. Error bars indicate SD; n = 20, and plants were prepared from at least five independent plants for each repeat. The means with ‘**’ represent significant differences from each other (*P*<0.01). Control, vector control plants; A2, A4, B1, C1.1, C2.1 represent the *GmHsp90A2*, *GmHsp90A4*, *GmHsp90B1*, *GmHsp90C1*.1 and *GmHsp90C2*.1 transgenic lines, respectively.

Three-week-old transgenic plants were treated with heat, salt and osmotic stress, respectively (Fig. 5), and their growth rates (measured as fresh weight) were significant differ from control plants (Fig. 6a, c). When treated with heat, salt or osmotic stress, the fresh weights of control plants were decreased to about 38% (heat stress), 92% (salt stress) or 43%(osmotic stress) compared with normal condition; while the fresh weights of transgenic plants were decreased less serious (Fig. 6a, c). In addition, the pod setting percentages of these plants were also different under heat stress. In normal conditions the transgenic and control plants showed no obvious differences in pod setting percentages; under heat stress, the pod setting percentage of control plants decreased to 52% while transgenic plants were better (Fig. 6b). These results indicated overexpressing GmHsp90 can decreased the damage of abiotic stresses and maintained the growth and development of Arabidopsis.

### Chlorophyll Content and Lipid Peroxidation Levels in the Transgenic Plants

**Figure 7:**
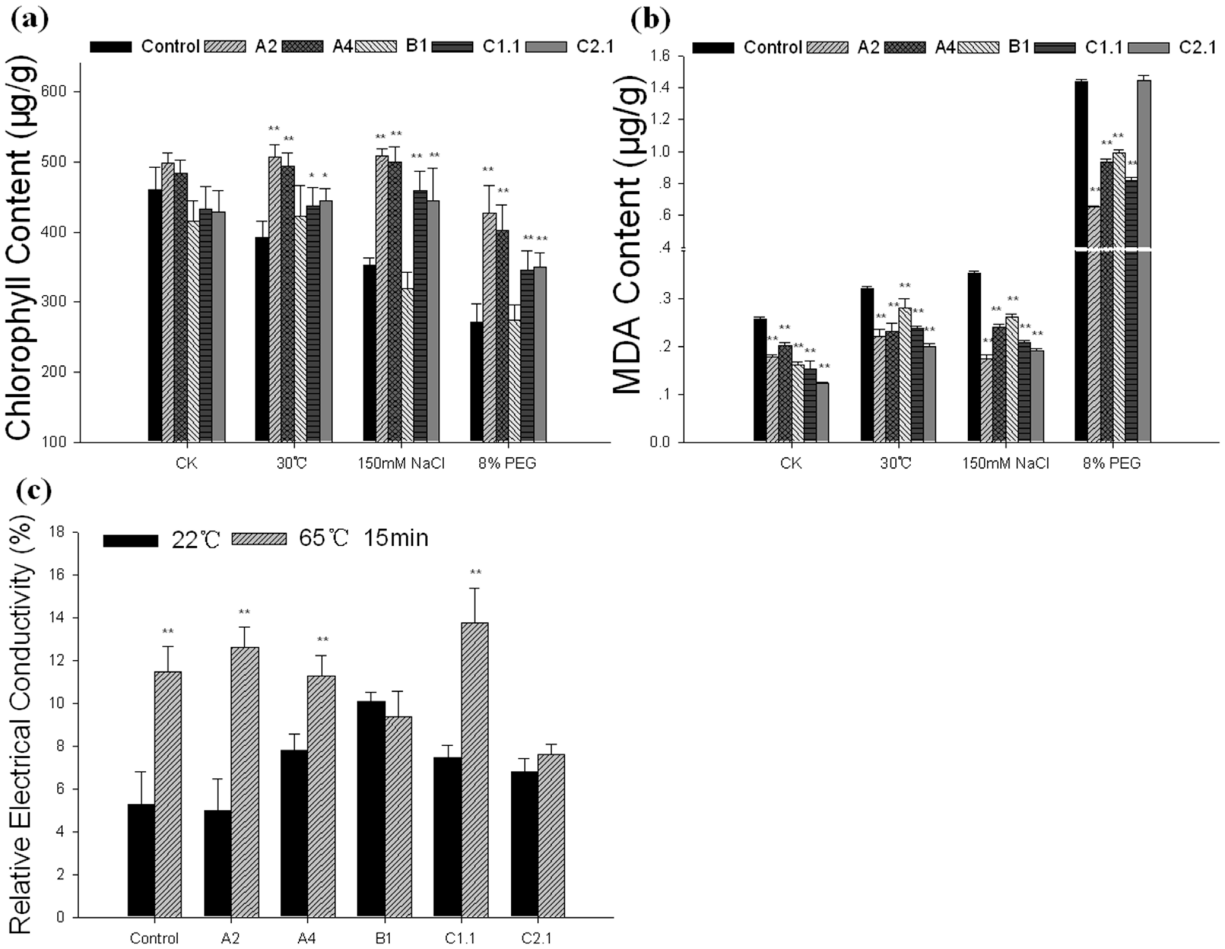
Physiological traits of transgenic Arabidopsis plants under abiotic stresses. Three-week-old seedlings were saturated with water (control), PEG (8%) or NaCl (150 mM) respectively for 3 d and recovered for 5 d. For heat stress, three-week-old seedlings were moved to 30°C until pod setting. Rosette leaf samples were taken at 3 d after treatment. The leaf discs of three-week-old transgenic and control plants were saturated with water and exposed to a temperature of 65°C for 15 min, and then relative electrical conductivity was measured. (a) Chlorophyll content of Arabidopsis under heat, salt and osmotic stress. (b) MDA content of Arabidopsis under heat, salt and osmotic stress. (c) Relative electrical conductivity of Arabidopsis under heat stress. Error bars indicate SD; n = 3; and leaves were prepared from at least five independent plants for each repeat. The means with ‘**’ represent significant differences from each other (*P*<0.01). The means with ‘*’ represent significant differences from each other (*P*<0.05).Control, vector control plants; A2, A4, B1, C1.1, C2.1 represent the *GmHsp90A2*, *GmHsp90A4*, *GmHsp90B1*, *GmHsp90C1*.1 and *GmHsp90C2*.1 transgenic lines, respectively.

To determine the reason of the decreased damage in GmHsp90 transgenic Arabidopsis plants under abiotic stresses, several researches were done and finally a change of chlorophyll content and lipid peroxidation levels were found. As we all known, stress would cause chlorophyll loss in plants; When treated with abiotic stresses, chlorophyll contents of control plants were decreased significantly higher than transgenic plants except for *GmHsp90B1* (Fig. 7a). In addition, the lipid peroxidation levels (measured as MDA content) of control plants were also higher than transgenic plants under abiotic stresses (Fig. 7b). However, MDA content of *GmHsp90C2.1* were higher than other transgenic plants corresponding to the less growth rate under osmotic stress (Fig. 6c, 7b). These results indicated overexpressing *GmHsp90A2*, *GmHsp90A4* and *GmHsp90C1.1* could prevent chlorophyll loss and decrease lipid peroxidation levels in Arabidopsis which may contribute to decrease damage of abiotic stresses and maintain the growth and development of Arabidopsis.

As mild heat stress did not cause severe damage to transgenic Arabidopsis plants in a short time, we tested a electrolyte leakage with an instant and extreme temperature as 65°C for 15 min. Interestingly, there were no significant differences in electrolyte leakage (measured as relative electrical conductivity) in *GmHsp90B1* and *GmHsp90C2.1* transgenic plants before and after heat shock while GmHsp90A and GmHsp90C1 were similar to control plants (Fig. 7c) that were discrepant to chlorophyll and MDA content. This suggested GmHsp90s may play different roles in cope with stress.

### Proline Content was Abnormal in GmHsp90 Transgenic Plants

**Figure 8:**
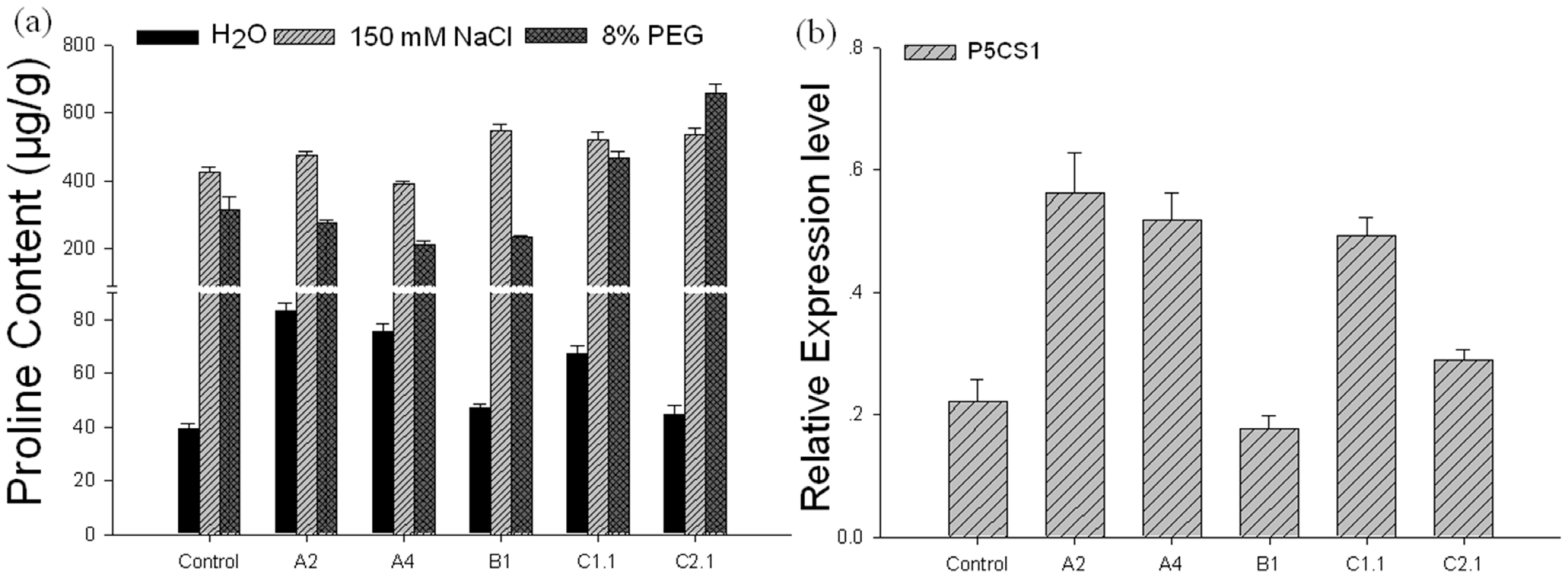
Proline content and quantitative real-time PCR analyses of relative expressions of *AtP5CS1* in transgenic Arabidopsis plants. For proline content, rosette leaf samples were taken simultaneous with other physiological traits tests. (a) Proline content of Arabidopsis plants, (b) Relative expressions of *AtP5CS1* in Arabidopsis plants. Error bars indicate SD; n = 3; and leaves were prepared from at least five independent plants for each repeat. For quantitative real-time PCR analyses, Arabidopsis plants were grown in normal conditions and rosette leaf samples were collected from three-week-old plants. An Arabidopsis *actin* gene was used as internal control for normalization. The relative mRNA level for each gene was calculated as ΔΔC_T_ values. The result of expression was generated by SigmaPlot 9.0. The transcript levels (means ± SD) displayed were each calculated using the qRT-PCR results of three technical repeats. Leaves of each repeat were prepared from at least five independent plants. Control, vector control plants; A2, A4, B1, C1.1, C2.1 represent the *GmHsp90A2*, *GmHsp90A4*, *GmHsp90B1*, *GmHsp90C1*.1 and *GmHsp90C2*.1 transgenic lines, respectively.

When measured the proline accumulation which is believed to play adaptive roles in plant stress tolerance [55] in transgenic Arabidopsis plants, an interesting phenomenon was found – proline accumulation in GmHsp90A and GmHsp90C1 was higher under normal conditions than other plants (Fig. 8a). Thus we investigated transcript levels of *AtP5CS1* encoding pyrroline- 5- carboxylate synthases 1, an important synthases in proline synthesis [55], in GmHsp90 overexpressing Arabidopsis plants in normal conditions. As expected, the transcript levels of *AtP5CS1* rose in *GmHsp90A2*, *GmHsp90A4* and *GmHsp90C1.1* overexpressing plants (Fig. 8b), which was correlated with their proline content. Interestingly, when searching http://bar.utoronto.ca/interactions/cgi-bin/arabidopsis_interactions_viewer we found that AtHsp90-1 and P5CS1 interact. This indicated some interesting correlations among Hsp90, P5CS1 and proline.

## Discussion

### Hsp90 are Highly Conserved in Soybean

Twelve members of the Hsp90 family were identified in soybean. Hsp90 from soybean are highly conserved (Fig. 1, 2a) and for ease of study, we used a new nomenclature system (Table 1) [24]. Like Hsp90 in Arabidopsis, soybean GmHsp90A3–GmHsp90A6 protein sequences were highly similar suggesting they may be functionally redundant. Although GmHsp90A1 and GmHsp90A2 were also highly homology to other GmHsp90As, they were a little different to others (Fig. 2a) and according to their expression under stresses; they may belong to an inducible form as already identified in mammals [24], [56]. Thus, GmHsp90A can also be defined as inducible and relatively constitutively expressed.

### The Expression Pattern of GmHsp90 Genes in Soybean Differs

In the present study, the expression patterns of GmHsp90 genes were diverse in various tissues and were most abundant in leaves (Fig. 2b), so we speculate that GmHsp90 genes may play different roles in soybean growth and development processes.

Almost all GmHsp90 genes can rapidly and strongly respond to heat stress except *GmHsp90C1* (Fig. 3 c). As GmHsp90s can rapidly accumulate and be maintained for a long period, they may play an important role in protecting plants, being involved in signal transduction or maintaining cell viability over a long period of heat stress [1]. The expressions of *GmHsp90A1* and *GmHsp90A2* were extremely high and remained at higher levels than other genes in the whole process, suggesting their inducible and crucial role in heat stress. The peak of GmHsp90C1 genes was delayed and indicated a possible specific function or different control mode under heat stress.

Most GmHsp90 genes also responded to osmotic and salt stress. The response to osmotic stress was rapid but not as strong as to heat stress while in salt stress the responses were obviously delayed and the peaks appeared by 24 h. We considered Hsp90 genes were involved in different pathways when participating in the response to salt compared with heat and osmotic stress. The transcript levels of GmHsp90Bs and GmHsp90C1 were lower than other induced genes, suggesting that they may not play a key role in osmotic and salt stress; while the expressions of *GmHsp90A1* and *GmHsp90A2* indicated an important role in abiotic stresses.

Interestingly, there were two or more copies in every group of Hsp90 genes but there were slight differences between the copies, suggesting that the major gene or complementary functions may have differentiated during the long evolutionary process.

### Overexpression of GmHsp90 Affects Growth, Development and Physiological Traits of Arabidopsis Through Different Pathways

In this study, overexpression of five GmHsp90 genes conferred Arabidopsis with different growth, development and physiological traits under abiotic stresses which may due to their discrepancy in structure, homology and locations.

In the present study, overexpression of GmHsp90A genes conferred higher germination rates and maintained the growth and development of Arabidopsis to abiotic stresses, especially *GmHsp90A2*, through decreasing chlorophyll loss and lipid peroxidation levels. As GmHsp90As had high homology to other cytoplasmic Hsp90 (Fig. 2a, S1a), we considered they may have similar function. Cytoplasmic Hsp90 function is a key component underlying maintenance of cellular homeostasis in the face of environmental fluctuation [57] and plays a role via cross-talk with other mechanisms and functions synergistically with other components to decrease cellular damage [7]. In addition, as chlorophyll content and MDA content were closely related to oxidative stress which caused by ROS transiently produced by abiotic stresses [58], we speculate GmHsp90A may also participate in decreasing oxidative stress damages under abiotic stresses.

However, an interesting phenomenon was found in this study, when treated with instant and extreme temperature, there were no significant differences in relative electrical conductivity between the *GmHsp90A2* overexpressing plants and control (Fig. 7c). When search with http://www. bar. utoronto. ca [59] it shows the expression of *AtHsp90.1* is rapidly and strongly induced by heat stress and quickly decreased to the normal level while other AtHsp90s not. So we consider when treated with instant and extreme temperature, the effects of overexpressing *GmHsp90A2* was overlay by the endogenous *AtHsp90-1*. In contrast, overexpressing of other GmHsp90s is not completely overlay. However, when treated with long and mildly heat stress, the thermotolerance *GmHsp90A2* transgenic plant was enhanced (Fig. 6, 7) this may attribute to the especial expression pattern of *AtHsp90-1* which is similarly to *GmHsp90A1* and *GmHsp90A2* in soybean (Fig. 3a), so under the long time heat stress, the effects of *GmHsp90A2* appears.*GmHsp90A4*, the constitutively expressed Hsp90 gene, was apparently partly functionally different to *GmHsp90A2* under extreme stress which indicates inducible and constitutively expressed GmHsp90 genes are functionally different in response to heat stress. Among all GmHsp90s inducible GmHsp90 may be most effectively under stress such as GmHsp90A2 and the similar results were also found in *GmHsp90A2* transgenic soybean (Xu *et al*., unpublished).

GmHsp90C1 can also confer higher germination rates and maintain the growth and development in transgenic Arabidopsis by decreasing lipid peroxidation and preventing chlorophyll loss, similarly to *GmHsp90A2* (Fig. 6, 7), but their pathways may slightly differ. *GmHsp90C1* overexpressing plants can accumulate large amounts of proline while accumulation of proline in *GmHsp90A2* plants is lower (Fig. 8a).In addition, the major function of CR88 (AtHsp90.5) is in chloroplast biogenesis [60] and in cyanobacteria, HtpG (homologous of chloroplast Hsp90) plays a role in protection from oxidative stress [61]. As GmHsp90C1 are highly homology to chloroplast Hsp90 (Fig. 2a, S1b), we consider they may have similar function in protecting chloroplasts which is a most injured organelle by oxidative stress under abiotic stresses.

In the present study, overexpression of *GmHsp90C2*.*1* did not confer Arabidopsis as high tolerance (fresh weight) as other transgenic Arabidopsis plants under osmotics stress (Fig. 6i, 7b). As GmHsp90C2 are highly homology to mitochondria Hsp90, they may have similar function but functions distinct from other Hsp90 [62].

In the present study, there was no significant difference in some performances of *GmHsp90B1* and control plants under abiotic stresses (Fig. 4b, c, 7a). GmHsp90B has high homology to other ER Hsp90 chaperone (Fig. 2a, S1a) which suggests it may function similar to ER Hsp90. Hsp90B as an ER chaperone has evolved to optimize the output of properly folded secretory and membrane proteins but its function is rather selective and interacts with late folding intermediates [63]. This may partly explain the performances of *GmHsp90B1* transgenic Arabidopsis plants.

Previously, researchers showed that overexpression of AtHsp90 in Arabidopsis could shift the equilibrium of Hsp90s with their client-bound states and by which impair plant tolerance to abiotic stresses [17]. In the present study, when the GmHsp90 transgenic plants grew in normal conditions, fresh weight was less vigorous than controls (Fig. 6a, c) which may be caused by disruption to homeostasis of Hsp90. However, when plants were treated with various stresses, their growths were better than control plants (Fig. 6). The adverse outcomes caused by disrupted homeostasis seemed to be offset.

The tolerances of *GmHsp90B1* and *GmHsp90C2.1* overexpressing plants under abiotic stresses were lower than other transgenic plants (Fig. 7a, b); however, when treated with extreme temperature they showed better tolerance (Fig. 7c). This indicates an interesting functional cooperation among the Hsp90s under different stresses. Further investigations of their cooperation under extreme stress are required.

Overexpressing GmHsp90 in Arabidopsis affects the synthesis and response system of proline.

In the present study, proline accumulation and the transcript level of *AtP5CS1* in GmHsp90A and GmHsp90C1 transgenic plants were higher under normal condition (Fig. 8a). P5CS is the rate-limiting enzyme in proline biosynthesis in plants and is subject to feedback inhibition by proline [64]. In addition, the loss of feedback regulation can be caused by conformational change to P5CS and result in a higher expression level of *P5CS* and so also proline accumulation [64] – similar to our results under normal conditions (Fig. 8). So we speculate overexpressing some GmHsp90s in Arabidopsis disturb the equilibrium of this feedback inhibition system through change in the conformation of P5CS. While under stress, the feedback regulation of P5CS is lost [64] so the accumulation of proline is not so discrepancy as in the normal condition (Fig. 8a).

When under osmotic stresses, there was a significant accumulation of proline in all plants but was lower in GmHsp90A overexpressing plants (Fig. 8a). We speculate that overexpressing GmHsp90A in Arabidopsis may affect the synthesis and response system of proline, slow the synthesis rate and finally decrease the accumulation of proline by two reasons: (1) there is already a higher accumulation of proline in GmHsp90A overexpressing plants and the ‘surplus’ proline is enough to cope with stress in the earlier stage; and (2) excessive Hsp90 proteins in the transgenic plants may replace the function of proline who can also act as ‘ chemical chaperones’ [65] in a short time. Here we surmise that there may be no significant boundary between some molecular chaperones and osmoprotectants – sometimes they may be functional analogous.

## Supporting Information

Figure S1 **Phylogenetic relationships analysis of HSP90 proteins in multiple species.** Phylogenetic trees were constructed with HSP90 protein sequences from *Glycine max* (GmHsp90), *Arabidopsis thaliana* (AtHsp90), *Drosophila melanogaster* (NP_523899.1, NP_651601.1), *Caenorhabditis elegans* (NP_506626.1), *Gallus gallus* (CAA30251.1, NP_989620.1), *Cryptococcus neoformans* (XP_568451.1), *Saccharomyces cerevisiae* (AAA02743.1), *Danio rerio* (AAH63951.1), *Oryza sativa* (BAA90487.1), *Chlamydomonas reinhardtii* (AAU10511.1), *Bacillus subtilis* (NP_391861.1), *Escherichia coli* (NP_415006.1), *Streptomyces coelicolor* (NP_631561.1),, *Pinus taeda* (ABV21762.1) and *Medicago truncatula* (XP_003589505.1). The trees were constructed using the neighbor-joining algorithm included in the MEGA4.0. software. (a) Phylogenetic relationships analysis of HSP90A and HSP90B proteins in multiple species. (b) Phylogenetic relationships analysis of HSP90C and HTPG proteins in multiple species.
(TIF)

Figure S2 **Expression of GmHsp90 genes in control and homozygous transgenic lines.** Transgenic Arabidopsis were generated by floral dip and transformants were selected for on 1/2 MS medium containing 20 mg/L Basta and then tested by PCR. Seeds from each T_1_ plant were individually collected and selected T_2_ plants were propagated and T_3_ or T_4_ homozygous overexpression lines were confirmed by RT-RCP analysis, and were used for further study.
(TIF)

Table S1 **Primers used to isolate the GmHsp90 genes.**
(DOC)

Table S2 **Amino acid similarity (%) between the GmHsp90.**
(DOC)

Table S3 **Primers used for quantitative real-time PCR.**
(DOC)

Table S4 **Primers used to construct of GmHsp90 plant expression vector.**
(DOC)
