# Supplementary figures and images for: Overexpression of GmHsp90s, a Heat Shock Protein 90 (Hsp90) Gene Family Cloning from Soybean, Decrease Damage of Abiotic Stresses in Arabidopsis thaliana
Source: PLoS One. 2013 Jul 25;8(7):e69810. doi: 10.1371/journal.pone.0069810 (PMC3723656; doi:10.1371/journal.pone.0069810)

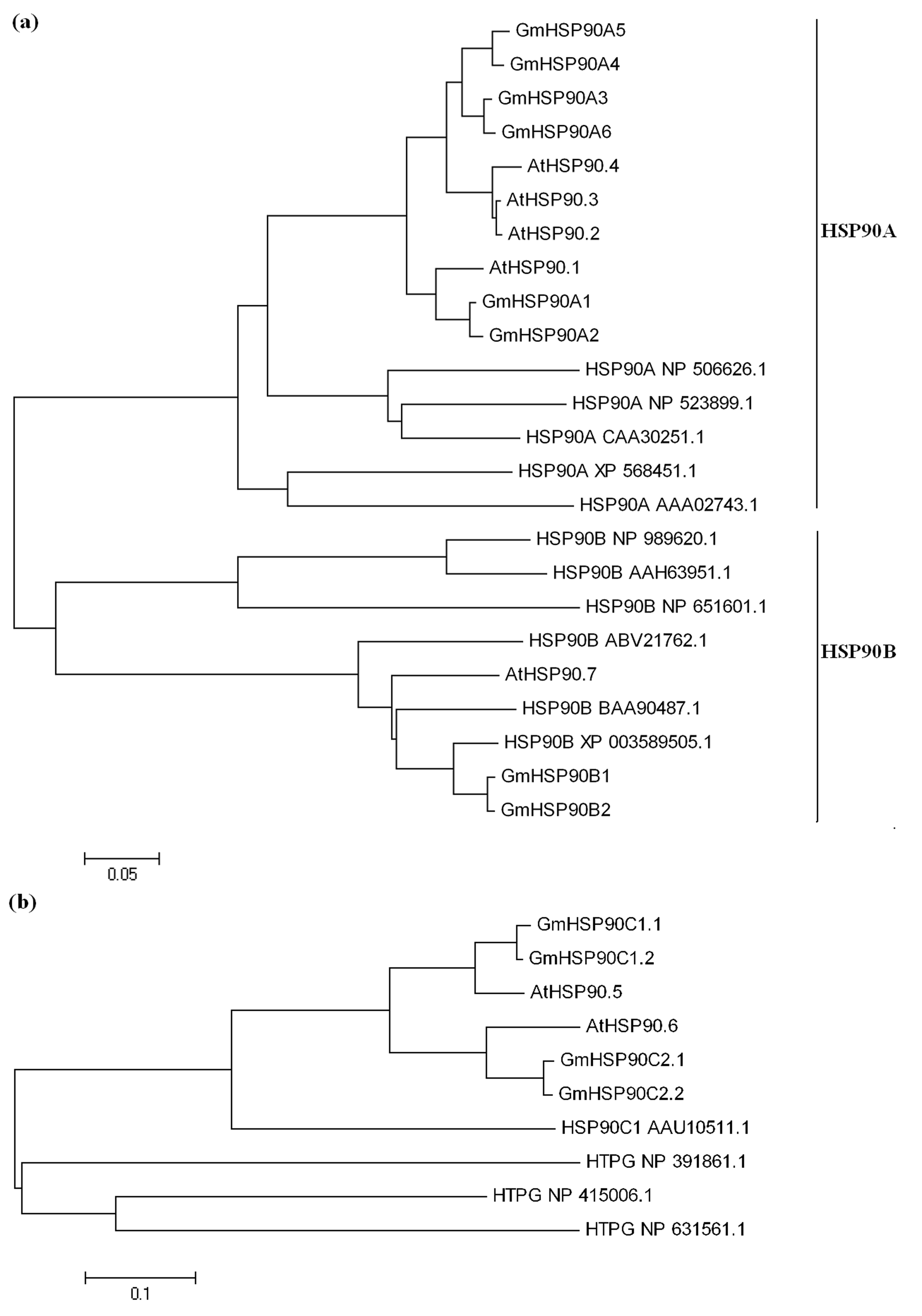

Supplement: Figure S1 — Phylogenetic relationships analysis of HSP90 proteins in multiple species. Phylogenetic trees were constructed with HSP90 protein sequences from Glycine max (GmHsp90), Arabidopsis thaliana (AtHsp90), Drosophila melanogaster (NP_523899.1, NP_651601.1), Caenorhabditis elegans (NP_506626.1), Gallus gallus (CAA30251.1, NP_989620.1), Cryptococcus neoformans (XP_568451.1), Saccharomyces cerevisiae (AAA02743.1), Danio rerio (AAH63951.1), Oryza sativa (BAA90487.1), Chlamydomonas reinhardtii (AAU10511.1), Bacillus subtilis (NP_391861.1), Escherichia coli (NP_415006.1), Streptomyces coelicolor (NP_631561.1),, Pinus taeda (ABV21762.1) and Medicago truncatula (XP_003589505.1). The trees were constructed using the neighbor-joining algorithm included in the MEGA4.0. software. (a) Phylogenetic relationships analysis of HSP90A and HSP90B proteins in multiple species. (b) Phylogenetic relationships analysis of HSP90C and HTPG proteins in multiple species. (TIF) [file pone.0069810.s001.tif]

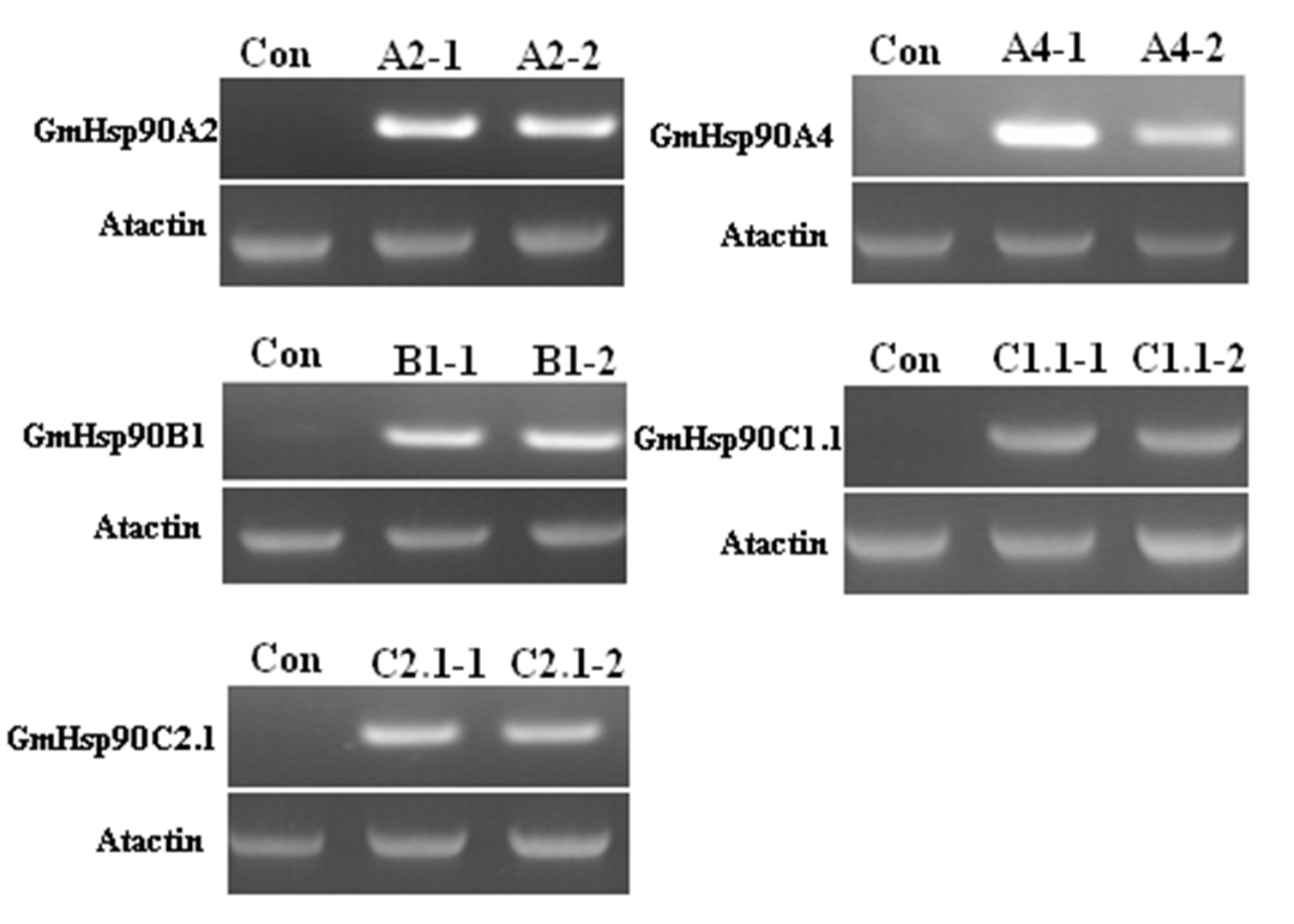

Supplement: Figure S2 — Expression of GmHsp90 genes in control and homozygous transgenic lines. Transgenic Arabidopsis were generated by floral dip and transformants were selected for on 1/2 MS medium containing 20 mg/L Basta and then tested by PCR. Seeds from each T1 plant were individually collected and selected T2 plants were propagated and T3 or T4 homozygous overexpression lines were confirmed by RT-RCP analysis, and were used for further study. (TIF) [file pone.0069810.s002.tif]
