# Supplementary material for: Overexpression of GmHsp90s, a Heat Shock Protein 90 (Hsp90) Gene Family Cloning from Soybean, Decrease Damage of Abiotic Stresses in Arabidopsis thaliana
Source: PLoS One. 2013 Jul 25;8(7):e69810. doi: 10.1371/journal.pone.0069810 (PMC3723656; doi:10.1371/journal.pone.0069810)
Supplement: Table S1 — Primers used to isolate the GmHsp90 genes. (DOC) [file pone.0069810.s003.doc]

Table S1. Primers used to isolate the GmHsp90 genes

| Gene | Locus ID | Sequence of primer pair (5’-3’) |
| --- | --- | --- |
| *GmHsp90*-3 | Glyma01g09310 | CTGTTTTGTGTTCTAACAATGGCT/GATTTGTAACTTATTCTATGAGGGCA |
| *GmHsp90*-4 | Glyma14g40320 | TAGAACACTAGTTTGTTGAGCACTG/TGTCATGCAAAATCTACAACTCG |
| *GmHsp90*-5 | Glyma16g29750 | TCTGCGGTTATCTTTTTGAGC/GGGTAACACGAGACCTTTGG |
| *GmHsp90*-6 | Glyma02g47580 | TTCTCAATCTCTCTCTGTCGTAGTT/ AAAAATGTCCACATGACTGTCC |
| *GmHsp90*-7 | Glyma02g47210 | CTCCTTCTGTCCGCTGCGACTCTCT/GGGCTAAAAACACAAAGGCAAATCA |
| *GmHsp90*-8 | Glyma08g44590 | TCGTTGTTCTCCCTCG /CCACAAGAAACGCATCATA |
| *GmHsp90-9* | Glyma14g01530 | CTACCTTCCCTCTTTTTCCGAC/CACCTAACTAGCCAACGTTCCAA |
| *GmHsp90*-10 | Glyma09g24410 | AACTTCGATTCTCTGCAATTCACTC/CCCCAACAAAACAAGGAAGATAAAGAC |
| *GmHsp90*-11 | Glyma02g13790 | TTTTTGTCTAACTATGGCTCCTGTG/ GGTTCTATGAGAACATAGTATGCCTAA |
| *GmHsp90*-12 | Glyma17g37820 | CGATGAGGAAGTGGACGG/GAAACCATGAAAAACAAAGGG |
| *GmHsp90*-13 | Glyma18g08220 | CTAAACCCTAAACGACTCTATCTCTC /GAAAAAACATAGCCAACTCCG |
| *GmHsp90*-14 | Glyma14g01100 | CCCTTCTAAAACCCTACTACC /AAGAAAGAACAGAAAGTAACGC |
