# Supplementary material for: Overexpression of GmHsp90s, a Heat Shock Protein 90 (Hsp90) Gene Family Cloning from Soybean, Decrease Damage of Abiotic Stresses in Arabidopsis thaliana
Source: PLoS One. 2013 Jul 25;8(7):e69810. doi: 10.1371/journal.pone.0069810 (PMC3723656; doi:10.1371/journal.pone.0069810)
Supplement: Table S2 — Amino acid similarity (%) between the GmHsp90. (DOC) [file pone.0069810.s004.doc]

Table S2. Amino acid similarity (%) between the GmHsp90s

|  | GmHSP  90A5 | GmHSP  90A3 | GmHSP  90A1 | GmHSP  90A4 | GmHSP  90A2 | GmHSP  90C1.1 | GmHSP  90B1 | GmHSP  90C1.2 | GmHSP  90C2.1 | GmHSP  90B2 | GmHSP  90A6 | GmHSP  90C2.2 |
| --- | --- | --- | --- | --- | --- | --- | --- | --- | --- | --- | --- | --- |
| GmHSP90A5 | 100 |  |  |  |  |  |  |  |  |  |  |  |
| GmHSP90A3 | 94.00 | 100 |  |  |  |  |  |  |  |  |  |  |
| GmHSP90A1 | 87.90 | 88.60 | 100 |  |  |  |  |  |  |  |  |  |
| GmHSP90A4 | 98.10 | 95.00 | 88.60 | 100 |  |  |  |  |  |  |  |  |
| GmHSP90A2 | 87.20 | 87.60 | 98.60 | 87.90 | 100 |  |  |  |  |  |  |  |
| GmHSP90C1.1 | 44.90 | 45.10 | 45.40 | 44.60 | 44.70 | 100 |  |  |  |  |  |  |
| GmHSP90B1 | 51.10 | 49.90 | 52.40 | 50.90 | 52.00 | 44.20 | 100 |  |  |  |  |  |
| GmHSP90C1.2 | 44.60 | 45.20 | 45.60 | 44.40 | 44.80 | 97.10 | 44.40 | 100 |  |  |  |  |
| GmHSP90C2.1 | 44.10 | 44.30 | 45.30 | 44.00 | 45.00 | 65.60 | 43.70 | 65.80 | 100 |  |  |  |
| GmHSP90B2 | 51.60 | 50.20 | 52.60 | 51.20 | 52.20 | 44.30 | 97.40 | 44.50 | 43.60 | 100 |  |  |
| GmHSP90A6 | 93.80 | 98.40 | 88.80 | 94.80 | 88.10 | 45.00 | 50.70 | 45.10 | 44.20 | 51.00 | 100 |  |
| GmHSP90C2.2 | 44.70 | 44.80 | 46.00 | 44.70 | 45.70 | 65.90 | 44.20 | 66.10 | 95.20 | 44.00 | 44.70 | 100 |
