# Supplementary material for: Overexpression of GmHsp90s, a Heat Shock Protein 90 (Hsp90) Gene Family Cloning from Soybean, Decrease Damage of Abiotic Stresses in Arabidopsis thaliana
Source: PLoS One. 2013 Jul 25;8(7):e69810. doi: 10.1371/journal.pone.0069810 (PMC3723656; doi:10.1371/journal.pone.0069810)
Supplement: Table S3 — Primers used for quantitative real-time PCR. (DOC) [file pone.0069810.s005.doc]

Table S3. Primers used for quantitative real-time PCR

| **Gene** | **sequence of primer pairs (5'-3')** |
| --- | --- |
| *GmHsp90A1* | TGATTTGCTCAGATACCACTCG/TGCATCCACCATGAAAAGAAC |
| *GmHsp90A2* | AGGCCACCCTTTGATTTGTTT/GGGCAAGTCATCGGAGTCG |
| *GmHsp90A3* | GACTGGGAAGAGCATTTGGC/ AGACACGGCGGACATAAAGC |
| *GmHsp90A4* | CAGAGTTGTTCATTCACATTATTCC/ TAGAAACCAACACCGAACTGC |
| *GmHsp90A5* | TCTAACTTCTGGGTTCAGCCTTG/TCAGCATCGGCATCAGCAT |
| *GmHsp90A6* | CAGCTAAGGAAGCGTGCGG/CTTCAGCTGCATCCTCGTCG |
| *GmHsp90B1* | TGAGATCAATCCTAGGCACCC/CTTGACATCATTATCATCGTTAACAG |
| *GMHSP90B2* | ACTCTGTTTATCTCGTGGCTGAC/TGTAATCTAATCTCAGTTCCACGC |
| *GmHsp90C1*.1 | AAATCATAAGCGCATTACACCAC/CTTTCGCCACCTTGTCACC |
| *GmHsp90C1*.2 | CAATTTGGTGTTTGATTCTACTCG/TTCCACCTCCACAGTTCTTGAC |
| *GmHsp90C2.1* | TACTCGCTCTTCGTCCGTCT/GCTTTGCTGTCATTTCCACC |
| *GmHsp90C2.2* | ACTACTCGCTCTGCCTTG/GTCATTTTCACCCACCTGT |
| *Gmtubulin* | GGAGTTCACAGAGGCAGAG/CACTTACGCATCACATAGCA |
| *AtP5CS1* | TGTGTGTTTGTGTATTTGGTTGAGAC/TGAGTACTAAGCAGAGAGGAAACAAAA |
| *Atactin* | GGAAAGGATCTGTACGGTAAC/TGTGAACGATTCCTGGAC |
