# Supplementary material for: Overexpression of GmHsp90s, a Heat Shock Protein 90 (Hsp90) Gene Family Cloning from Soybean, Decrease Damage of Abiotic Stresses in Arabidopsis thaliana
Source: PLoS One. 2013 Jul 25;8(7):e69810. doi: 10.1371/journal.pone.0069810 (PMC3723656; doi:10.1371/journal.pone.0069810)
Supplement: Table S4 — Primers used to construct of GmHsp90 plant expression vector. (DOC) [file pone.0069810.s006.doc]

Table S4. Primers used to construct of GmHsp90 plant expression vector and transgenic plants detection

| **Gene** | **sequence of primer pairs (5'-3')** |
| --- | --- |
| *GmHsp90A2* | GGGGACAAGTTTGTACAAAAAAGCAGGCTTCATGGCGGACGCTGAAACC/GGGGACCACTTTGTACAAGAAAGCTGGGTCGTCCACTTCCTCCATCTT |
| *GmHsp90A4* | GGGGACAAGTTTGTACAAAAAAGCAGGCTTCATGGCTTCGGAGACTGAG/GGGGACCACTTTGTACAAGAAAGCTGGGTCATCAACTTCTTCCATCTT |
| *GmHsp90B1* | GGGGACAAGTTTGTACAAAAAAGCAGGCTTCATGAGGAAGTGGACGGTT/GGGGACCACTTTGTACAAGAAAGCTGGGTCCAACTCGTCCTTGACATC |
| *GmHsp90C1*.*1* | GGGGACAAGTTTGTACAAAAAAGCAGGCTTCATGGCTCCTGTGCCGAGC/GGGGACCACTTTGTACAAGAAAGCTGGGTCATCAGTTGTCCATGGATC |
| *GmHsp90C2.1* | GGGGACAAGTTTGTACAAAAAAGCAGGCTTCATGCTGCAGAGGCTCTCT/GGGGACCACTTTGTACAAGAAAGCTGGGTCCTTCTGACCACCAGCCTC |

Black underscore represents *att*B sites
